# Vaccine effectiveness against severe acute respiratory infections (SARI) COVID-19 hospitalisations estimated from real-world surveillance data, Slovenia, October 2021

**DOI:** 10.2807/1560-7917.ES.2022.27.1.2101110

**Published:** 2022-01-06

**Authors:** Marta Grgič Vitek, Irena Klavs, Veronika Učakar, Mojca Serdt, Maja Mrzel, Marjana Vrh, Mario Fafangel

**Affiliations:** 1 Communicable Diseases Centre, National Institute of Public Health, Ljubljana, Slovenia

**Keywords:** vaccine effectiveness, mRNA vaccines, viral vector vaccines, severe acute respiratory infection (SARI), COVID-19, surveillance, Slovenia

## Abstract

We estimated vaccine effectiveness (VE) against severe COVID-19 during October 2021, using Slovenian surveillance data. For people fully vaccinated with any vaccine in age groups 18–49, 50–64, ≥ 65 years, VE was 86% (95% CI: 79–90), 89% (85–91), and 77% (74–81). Among ≥ 65 year-olds fully vaccinated with mRNA vaccines, VE decreased from 93% (95% CI: 88–96) in those vaccinated ≤ 3 months ago to 43% (95% CI: 30–54) in those vaccinated ≥ 6 months ago, suggesting the need for early boosters.

To inform vaccination strategy it is important to understand the effectiveness of vaccination against severe coronavirus disease (COVID-19). In Slovenia, by the end of September 2021, the vaccination coverage against COVID-19 was rather low (47.2% among 18–49 year-olds, 63.5% among 50–64 year-olds, and 76.1% among ≥ 65 year-olds) and waning immunity may have occurred, especially among elderly people  (≥ 65 year-olds) who were vaccinated first [1]. Here we estimated vaccine effectiveness (VE) against hospitalisation due to severe acute respiratory infection (SARI) COVID-19 during October 2021 (weeks 39 to 43; from 27 September to 31 October), when the severe acute respiratory syndrome coronavirus 2 (SARS-CoV-2) Delta variant was predominant, by age groups, for all vaccines used and separately for mRNA vaccines and viral vector vaccines. Additionally, we estimated VE against hospitalisation due to SARI COVID-19 for mRNA vaccines by time since vaccination.

We used comprehensive national COVID-19 surveillance data and data on vaccination against COVID-19 collected at the National Institute of Public Health (Nacionalni inštitut za javno zdravje (NIJZ)).

## Case definitions and other definitions

A SARI COVID-19 case was defined as an individual with SARI and a positive SARS-CoV-2 reverse transcription PCR (RT-PCR) or antigen test result at admission to hospital [2]. A case with a previous COVID-19 diagnosis was defined as an individual with a record of a positive SARS-CoV-2 RT-PCR in the national COVID-19 database more than 3 weeks before the week under observation. Fully vaccinated individuals were those who completed the primary recommended vaccination schedule with any vaccine used in Slovenia: Comirnaty (BNT162b2 mRNA, BioNTech-Pfizer, Mainz, Germany/New York, United States) or Spikevax (mRNA-1273, Moderna, Cambridge, United States) or Vaxzervia (ChAdOx1 nCoV-19, Oxford-AstraZeneca, Cambridge, United Kingdom) or Janssen vaccine (Ad26.COV2-S, Janssen-Cilag International NV, Beerse, Belgium) at least 14 days before the week under observation in the national EPISARI surveillance database. Unvaccinated individuals were defined as individuals who had not received any dose of vaccine against COVID-19.

## Data sources

Three different sources were used in the analysis: (i) weekly numbers of SARI COVID-19 cases admitted to hospitals were extracted from EPISARI [2], (ii) vaccination status of all individuals vaccinated in Slovenia was obtained from the national Electronic registry of vaccinated individuals and adverse events following immunisation (eRCO) [3] and (iii) previous diagnosis of COVID-19 was retrieved from the national COVID-19 database. By using unique national identifiers, we were able to link the data from the three databases to ascertain the vaccination status of SARI COVID-19 cases admitted to hospitals and previous diagnosis of COVID-19 among fully vaccinated individuals.

## Calculating vaccine effectiveness

To estimate VE against hospitalisation due to SARI COVID-19 for all vaccines used in Slovenia and separately for mRNA and viral vector vaccines (by age groups and by time since vaccination), we used the respective rates of SARI COVID-19 cases in fully vaccinated individuals without previous diagnosis of COVID-19 and respective rates of SARI COVID-19 cases in unvaccinated individuals without previous diagnosis of COVID-19.

Vaccine effectiveness was calculated using the formula: (1 − RR) × 100, where RR was the ratio of severe COVID-19 cases rate in the fully vaccinated, to the equivalent rate in the unvaccinated.

To obtain denominators for fully vaccinated individuals without previous diagnosis of COVID-19, the number of fully vaccinated individuals with previous COVID-19 diagnosis was subtracted from the number of fully vaccinated individuals. To obtain estimates of denominators for unvaccinated individuals without previous diagnosis of COVID-19, we subtracted the number of individuals who have received at least one dose of any vaccine against COVID-19, and the number of individuals with previous COVID-19 diagnosis, from the total number of individuals in the Central Population Registry as of 1 of January 2021.

## Vaccine effectiveness against SARI COVID-19 hospitalisations

During October 2021, VE for all vaccines as well as for mRNA and viral vector vaccines against SARI COVID-19 hospitalisations was lowest in the oldest age group (Table 1). Viral vector vaccines were less effective in preventing hospitalisations due to SARI COVID-19 in comparison to mRNA vaccines in all age groups.

**Table 1:** Vaccine effectiveness for all vaccine types, mRNA vaccines and viral vector vaccines, against hospitalisation due to SARI COVID-19, by age group, Slovenia, October 2021

Age group (years)	Fully vaccinated individuals without previous COVID-19^a,b^		Unvaccinated individuals without previous COVID-19^a,c^		Vaccine effectiveness	
	Number of SARI COVID-19 cases^d^	Rate per 100,000 population	Number of SARI COVID-19 cases^d^	Rate per 100,000 population	%	95% CI
All vaccines						
18–49	27	8.0	179	55.8	86	79–90
50–64	63	25.7	270	229.9	89	85–91
≥ 65	281	99.2	383	438.2	77	74–81
mRNA vaccines						
18–49	8	4.2	179	55.8	92	85–96
50–64	23	15.4	270	229.9	93	90–96
≥ 65	243	93.1	383	438.2	79	75–82
Viral vector vaccines						
18–49	19	13.2	179	55.8	76	62–85
50–64	39	41.3	270	229.9	82	75–87
≥ 65	36	169.2	383	438.2	61	46–73

CI: confidence interval; COVID-19: coronavirus disease; mRNA: messenger ribonucleic acid; SARI: severe acute respiratory infection; SARS-CoV-2: severe acute respiratory syndrome coronavirus 2.

^a^ Individuals without previous COVID-19 diagnosis were defined as individuals without a record of a positive SARS-CoV-2 RT-PCR in the national COVID-19 database more than 3 weeks before the week under observation within the SARI surveillance database known as EPISARI [2].

^b^ Fully vaccinated individuals with any vaccine were defined as individuals who had received two doses of mRNA vaccines (Comirnaty (BNT162b2 mRNA, BioNTech-Pfizer, Mainz, Germany/New York, United States) or Spikevax (mRNA-1273, Moderna, Cambridge, United States)) or viral vector vaccine (Vaxzervia (ChAdOx1 nCoV-19, Oxford-AstraZeneca, Cambridge, United Kingdom)) or one dose of viral vector vaccine Janssen (Ad26.COV2-S, Janssen-Cilag International NV, Beerse, Belgium) at least 14 days before the week under observation.

^c^ Unvaccinated individuals were defined as individuals who had not received any dose of vaccine against COVID-19.

^d^ SARI COVID-19 cases were defined as all SARI cases testing positive for SARS-CoV-2 by PCR or antigen test at admission to hospitals.

Data sources: EPISARI surveillance of severe acute respiratory infections within comprehensive COVID-19 surveillance [2], national electronic registry of vaccinated individuals and adverse events following vaccinations (eRCO), national COVID-19 dataset and Slovenian Central Population Registry.

Among individuals fully vaccinated with mRNA vaccines 6 months ago or longer, VE against hospitalisation due to SARS COVID-19 was substantially lower among those aged 65 years and older (43%; 95% CI: 31–54%) in comparison to those 50 to 64 years old (89%; 95% CI: 56–97%) (Table 2).

**Table 2:** Vaccine effectiveness of mRNA vaccines against hospitalisation due to SARI COVID-19 by time since vaccination and age group, Slovenia, October 2021

Age group (years)	Fully vaccinated individuals without previous COVID-19^a,b^		Unvaccinated individuals without previous COVID-19^a,c^		Vaccine effectiveness	
	Number of SARI COVID-19 cases^d^	Rate per 100,000 population	Number of SARI COVID-19 cases^d^	Rate per 100,000 population	%	95% CI
Vaccinated ≤ 3 months ago						
18–49	3	1.7	179	55.8	97	90–99
50–64	14	12.7	270	229.9	94	91–97
≥ 65	17	31.3	383	438.2	93	88–96
Vaccinated 4–5 months ago						
18–49	0	0.0	179	55.8	NA	NA
50–64	7	22.8	270	229.9	90	79–95
≥ 65	107	67.5	383	438.2	85	81–88
Vaccinated ≥ 6 months ago						
18–49	5	42.8	179	55.8	23	0–69
50–64	2	25.0	270	229.9	89	56–97
≥ 65	119	248.3	383	438.2	43	30–54

CI: confidence interval; COVID-19: coronavirus disease; mRNA: messenger ribonucleic acid; NA: not applicable; SARI: severe acute respiratory infection; SARS: severe acute respiratory syndrome coronavirus 2.

^a^ Individuals without previous COVID-19 diagnosis were defined as individuals without a record of a positive SARS-CoV-2 RT-PCR in the national COVID-19 database more than 3 weeks before the week under observation within the SARI surveillance database known as EPISARI [2].

^b^ Fully vaccinated individuals with MRNA vaccines were defined as individuals who had received two doses of Comirnaty (BNT162b2 mRNA, BioNTech-Pfizer, Mainz, Germany/New York, United States) or Spikevax (mRNA-1273, Moderna, Cambridge, United States) vaccine at least 14 days before the week under observation.

^c^ Unvaccinated individuals were defined as individuals who had not received any dose of vaccine against COVID-19.

^d^ SARI COVID-19 cases were defined as all SARI cases testing positive for SARS-CoV-2 by PCR or antigen test at admission to hospitals.

Data sources: EPISARI surveillance of severe acute respiratory infections within comprehensive COVID-19 surveillance [2], national electronic registry of vaccinated individuals and adverse events following vaccinations (eRCO), national COVID-19 dataset and Slovenian Central Population Registry.

### Ethical statement

Ethical approval was not necessary because all surveillance data used are mandatorily collected according to the law.

## Discussion

The effectiveness of COVID-19 vaccines has been recently intensively studied. Some reports suggested decreased VE against infection by the SARS-CoV-2 Delta variant [4], others indicated modest differences in VE against symptomatic disease with the Delta variant as compared with the Alpha variant [5], and some studies found maintained VE against hospitalisation due to severe disease [6-8]. Our results suggest that vaccines used in Slovenia were effective against hospitalisation due to SARI COVID-19 in fully vaccinated individuals during October 2021 when the Delta variant was predominant. The estimated VE was 86% (95% CI: 79–90), 89% (95% CI: 85–91), and 77% (95% CI: 74–81) among age groups 18–49, 50–64, and 65 years and older, for vaccination with any vaccine used in Slovenia. For mRNA vaccines the VE point percentage values showed an even greater risk reduction of SARI COVID-19 hospitalisation in all age groups, even though the 95% confidence intervals overlapped. Similar effectiveness of mRNA vaccines against hospitalisation due to severe COVID-19 was observed by others [6-9], and the European Centre for Disease Prevention and Control estimated the effectiveness of the Comirnaty vaccine against hospitalisation due to SARI-confirmed COVID-19 during the pre-Delta period in elderly people (≥ 65 years) at 91% (95% CI: 80–96) [10].

The estimated VE against hospitalisation due to SARI COVID-19 in fully vaccinated Slovenian individuals during October 2021 for vector vaccines was statistically significantly lower compared with mRNA VE for all three age groups investigated here.

It is important to note that VE of mRNA vaccines against SARI COVID-19 hospitalisation decreased substantially with the increasing time since vaccination, especially in elderly people (≥ 65 years) to only 43% (95% CI: 31–54%) for those fully vaccinated 6 months ago or more. This finding suggested that more than half of the elderly people fully vaccinated  6 months or more ago were no longer sufficiently protected against hospitalisation due to severe COVID-19. Waning immunity has also been reported by others [11-13]. Thus, our results provide additional evidence of substantially declining protection with time since vaccination against hospitalisation due to severe COVID-19 in elderly people and the need for a timely booster dose, especially in this age group.

The strength of our study is in the real-world national surveillance data collected from all Slovenian hospitals (EPISARI), and data from official national registries (eRCO and COVID-19 database). Surveillance of SARI COVID-19 cases within EPISARI has some limitations, however, which have been described in detail elsewhere [2]. In brief, the data collection process is at the discretion of the individual hospital and there may be variation in case ascertainment [2]. Some SARI cases and COVID-19 cases may have been misclassified or under-reported [2]. As previous infection with SARS-CoV-2 may confer protection for some time, it was important to exclude prior COVID-19 confirmed cases from analysis to not underestimate VE [14]. It should be noted that the likelihood of systematic unmeasured differences between vaccinated and unvaccinated individuals (e.g. adherence to non-pharmaceutical measures) could influence the rates of COVID-19 in the two groups. Also, comparison of VE between different age groups may be affected by differences in the overall time after full vaccination in different age groups. As at 31 October 2021, the average time elapsed since being fully vaccinated was 110 days for 18−49 year-olds, 131 days for 50−64 year-olds and 172 days for those aged 65 years and older. Finally, as no information on comorbidities of SARI COVID-19 cases was available it was difficult to assess to what extent the reduced VE among individuals immunised at the beginning of the pandemic occurred because individuals at high risk of severe disease were prioritised for early vaccination. For example, in the age group 18–49 years old, among five SARI COVID-19 cases fully vaccinated 6 months ago or longer, we were able to obtain information on comorbidities associated with high risk for severe COVID-19 for four individuals. Nevertheless, we believe that our real-world national VE data are robust enough to inform our vaccination strategies.

### Conclusion

Our results show that vaccines used in Slovenia were highly effective against hospitalisation due to SARI COVID-19 in fully vaccinated individuals up to 6 months after completed vaccination during the period of predominant Delta variant circulation. Six months or longer after completed vaccination, protection decreased substantially for those aged 65 years and older. It is thus important that individuals belonging to this group receive a booster dose as soon as possible at an interval of 6 months or even earlier after primary vaccination to prevent a substantial proportion of hospitalisations due to SARI COVID-19. We plan to assess changes in VE for mRNA vaccines currently used in the near future, when the Omicron variant will have become predominant in our country.

## References

[1] National institute of Public Health (NIJZ). Cepljenje proti COVID-19 v Sloveniji. [Vaccination against COVID-19 in Slovenia]. Ljubljana: NIJZ. [Accessed: 2 Dec 2021]. Slovenian. Available from: https://app.powerbi.com/view?r=eyJrIjoiYWQ3NGE1NTMtZWJkMi00NzZmLWFiNDItZDc5YjU5MGRkOGMyIiwidCI6ImFkMjQ1ZGFlLTQ0YTAtNGQ5NC04OTY3LTVjNjk5MGFmYTQ2MyIsImMiOjl9

[2] Klavs I Serdt M Učakar V Grgič-Vitek M Fafangel M Mrzel M Enhanced national surveillance of severe acute respiratory infections (SARI) within COVID-19 surveillance, Slovenia, weeks 13 to 37 2021. Euro Surveill. 2021;26(42):2100937. 10.2807/1560-7917.ES.2021.26.42.2100937 34676822 PMC8532507

[3] National institute of Public Health (NIJZ). Elektronski register cepljenih oseb in neželenih učinkov po cepljenju. [Electronic Registry of vaccinated individuals and adverse events following vaccinations]. Ljubljana: NIJZ. [Accessed: 2 Dec 2021]. Slovenian. Available from: https://www.nijz.si/sl/elektronski-register-cepljenih-oseb-in-nezelenih-ucinkov-po-cepljenju-erco

[4] Seppälä E Veneti L Starrfelt J Danielsen AS Bragstad K Hungnes O Vaccine effectiveness against infection with the Delta (B.1.617.2) variant, Norway, April to August 2021. Euro Surveill. 2021;26(35):2100793. 10.2807/1560-7917.ES.2021.26.35.2100793 34477054 PMC8414959

[5] Lopez Bernal J Andrews N Gower C Gallagher E Simmons R Thelwall S Effectiveness of Covid-19 vaccines against the B.1.617.2 (Delta) variant. N Engl J Med. 2021;385(7):585-94. 10.1056/NEJMoa2108891 34289274 PMC8314739

[6] Tenforde MW Self WH Naioti EA Ginde AA Douin DJ Olson SM Sustained Effectiveness of Pfizer-BioNTech and Moderna Vaccines Against COVID-19 Associated Hospitalizations Among Adults - United States, March-July 2021. MMWR Morb Mortal Wkly Rep. 2021;70(34):1156-62. 10.15585/mmwr.mm7034e2 34437524 PMC8389395

[7] Harder T Külper-Schiek W Reda S Treskova-Schwarzbach M Koch J Vygen-Bonnet S Effectiveness of COVID-19 vaccines against SARS-CoV-2 infection with the Delta (B.1.617.2) variant: second interim results of a living systematic review and meta-analysis, 1 January to 25 August 2021. Euro Surveill. 2021;26(41):2100920. 10.2807/1560-7917.ES.2021.26.41.2100920 34651577 PMC8518304

[8] Puranik A, Lenehan PJ, Silvert E, Niesen MJ, Corchado-Garcia J, O’Horo JC, et al. Comparison of two highly-effective mRNA vaccines for COVID-19 during periods of Alpha and Delta variant prevalence. medRxiv. 2021:2021.08.06.21261707v3. 10.1101/2021.08.06.21261707

[9] Thompson MG Stenehjem E Grannis S Ball SW Naleway AL Ong TC Effectiveness of Covid-19 Vaccines in Ambulatory and Inpatient Care Settings. N Engl J Med. 2021;385(15):1355-71. 10.1056/NEJMoa2110362 34496194 PMC8451184

[10] European Centre for Disease Prevention and Control (ECDC). Interim analysis of COVID-19 vaccine effectiveness against Severe Acute Respiratory Infection due to laboratory-confirmed SARS-CoV-2 among individuals aged 65 years and older, ECDC multi-country study. Stockholm: ECDC; 8 Oct 2021. Available from: https://www.ecdc.europa.eu/en/publications-data/interim-analysis-covid-19-vaccine-effectiveness-against-severe-acute-respiratory

[11] Israel A Merzon E Schäffer AA Shenhar Y Green I Golan-Cohen A Elapsed time since BNT162b2 vaccine and risk of SARS-CoV-2 infection: test negative design study. BMJ. 2021;375:e067873. 10.1136/bmj-2021-067873 34819275 PMC9083235

[12] Chemaitelly H, Tang P, Hasan MR, AlMukdad S, Yassine HM, Benslimane FM, et al. Waning of BNT162b2 Vaccine Protection against SARS-CoV-2 Infection in Qatar. N Engl J Med. 2021:NEJMoa2114114. 10.1056/NEJMoa2114114 PMC8522799 34614327

[13] Goldberg Y, Mandel M, Bar-On YM, Bodenheimer O, Freedman L, Haas EJ, et al. Waning Immunity after the BNT162b2 Vaccine in Israel. N Engl J Med. 2021:NEJMoa2114228. 10.1056/NEJMoa2114228 PMC8609604 34706170

[14] Havervall S, Ng H, Jernbom Falk A, Greilert-Norin N, Månberg A, Marking U, et al. Robust humoral and cellular immune responses and low risk for reinfection at least 8 months following asymptomatic to mild COVID-19. J Intern Med. 2021;joim.13387.; Epub ahead of print. 10.1111/joim.13387 PMC8661920 34459525

